# Research on U-shaped relationship between short-term debt for long-term use and supply chain enterprise default risk: Evidence from Chinese listed firms

**DOI:** 10.1371/journal.pone.0293284

**Published:** 2023-10-23

**Authors:** Huaqiao Shen, Jinlong Chen

**Affiliations:** School of Business Administration, Huaqiao University, Quanzhou, China; Universiti Malaysia Sabah, MALAYSIA

## Abstract

This paper empirically investigates the impact mechanism of short-term debt for long-term use and the default risk of supply chain firms with the data of Chinese A-share listed firms from 2007 to 2021. The study shows that there is a significant U-curve relationship between short-term debt for long-term use and supply chain firms’ default risk, and too high or too low a level of short-term loans and long-term investments will worsen firms’ default risk. In addition, firm performance plays an mediating effect in the process of short-term debt for long-term investment affecting the default risk of supply chain firms. Finally, customer effect and firm heterogeneity play a moderating role in the impact of short-term loans and long-term investments on the default risk of supply chain firms, and the U-shaped relationship will be strengthened under the high-intensity customer effect. This study has important theoretical and practical significance for analyzing the impact of default risk contagion in supply chain enterprises.

## 1. Introduction

On April 6, 2022, the People’s Bank of China issued the Financial Stability Law of the People’s Republic of China (Draft for public Comments), which clearly proposed to strengthen the prevention and defusing of financial and credit risks and steadily improve risk management. Meanwhile, the central government further pointed out that we should adhere to the macro strategic goals of preventing major risks and resolutely not touching the bottom line of systemic financial risks. To achieve the above macro-control goals, the micro-guarantee is to deal with the problem of short-term debt for long-term use of enterprises, and prevent and resolve the default risk of enterprises. At the same time, with the development of supply chain, although the integration and optimization of supply chain enterprises help to cope with the challenges of economic globalization, the increasingly dependent cooperative relationship of supply chain enterprises gradually constitutes an important factor of the vulnerability of supply chain system. The default risk of a certain actor in the supply chain can easily rely on the intermediary carriers such as material flow, information flow and financial flow in the supply chain network. The transmission spreads to the upstream and downstream enterprises of the supply chain, and eventually evolves into the default risk accident of the whole supply chain system, which hinders the development of the supply chain system and the economy. Therefore, it is of great theoretical and practical significance to correctly understand the influencing mechanism of the level of short-term debt for long-term use of enterprises on the default risk of supply chain enterprises, for preventing supply chain risk accidents and achieving macro goals such as financial risk regulation.

Short-term debt for long-term use refers to the investment and financing phenomenon in which enterprises continuously roll short-term debt to support long-term project investment [1]. Some scholars also call it "short-term loan for long investment" or "maturity mismatch of investment and financing". On the one hand, a certain degree of short-term debt for long-term use can provide working capital support for the investment of supply chain enterprises and alleviate the financing constraints of supply chain enterprises [2–4]. In addition, compared with long-term debt, the advantages of short-term loans, such as low interest rate and fast lending, are not only conducive to the reduction of corporate financing transaction costs [5], can also transmit positive signals to the external supply chain enterprises, further improve financing flexibility, and inhibit the default risk of supply chain enterprises to a certain extent; On the other hand, since the financial crisis and the outbreak of COVID-19, the long-term "financial repression" environment has made it difficult for most of China’s supply chain smes to get effective support for long-term investment and financing funds [6], which negatively improves the level of short-term debt for long-term use" of supply chain enterprises [7], and thus amplifies the short-term debt repayment pressure of enterprises. It causes liquidity risk and operational risk of supply chain enterprises [8, 9], which ultimately intensifies the default risk of enterprises [10]. However, all kinds of risk effects caused by the default risk of supply chain enterprises will be accumulated through the specific contagion mechanism and network structure of supply chain, amplified and even mutated in the form of "domino" effect, and spread to the whole supply chain network, which hinders the development of supply chain system and economy, and brings potential huge losses to the industry, the country and even the world economy. However, the existing studies have not provided in-depth empirical evidence on the economic consequences of short-term loans and long-term investments, and whether they can inhibit or promote the long-term stable development of enterprises, which need to be further explored. This paper investigates the impact of "short-term debt for long-term use" on enterprise risk, in order to further clarify the mechanism through which the mismatch of investment and financing term structure plays a role, which is helpful to supplement the research on the economic consequences of the mismatch of investment and financing term structure, and clarify how "short-term loan and long-term investment" affects enterprise development. Based on this, this paper takes the Shanghai and Shenzhen A-share listed enterprises from 2007 to 2021 as the research sample, through the construction of the "investment short-term loan" sensitivity model, objectively verifies the existence of "short-term debt long-term" in China’s supply chain enterprises, and empirically tests the relationship between short-term debt long-term and the default risk behavior of supply chain enterprises, as well as the role of financing constraints. In the current complex and changeable supply chain context, what impact will this investment and financing phenomenon of continuously rolling short-term debt support long-term project investment have on the default risk of supply chain enterprises? What mechanism affects the default risk of supply chain enterprises?

The main contributions of this paper are as follows: (1) Starting from the phenomenon of long term use of short bonds of micro enterprises, it proves from various angles that there is indeed a U-shaped relationship between short-term debt for long-term use and the default risk of supply chain enterprises, rather than a simple linear relationship, and makes important theoretical contributions to the relevant research on short-term debt for long-term use and the default risk of supply chain enterprises. (2) This study not only verifies the direct influence of the U-shaped relationship between the short-term debt for long-term use and the default risk of supply chain enterprises, but also examines the intermediary conduction effect and regulating mechanism between the two. (3) The research conclusions of this paper enrich the relevant studies on the short-term debt for long-term use and the default risk of supply chain enterprises, and provide new evidence on how to reasonably optimize the short-term debt for long-term use of supply chain enterprises. Under the background of frequent default events of supply chain enterprises and incomplete financial system, the influence of the short-term debt for long-term use on the default risk of supply chain enterprises is analyzed. It plays an important role in arranging financing structure reasonably for supply chain enterprises, strengthening risk control, preventing default risk and maintaining the safety and stability of China’s financial system.

## 2. Theoretical analysis and research hypothesis

The matching principle of investment and financing term, as its name implies, requires supply chain enterprises to consider the matching principle of investment term and debt maturity structure when conducting investment and financing activities [11]. Generally speaking, supply chain enterprises prefer prudent investment to avoid the problem of high liquidity risk caused by the term structure of aggressive investment and financing. However, in the practice of investment and financing of supply chain enterprises in China, they are troubled by financial constraints such as financing difficulties, high financing costs and financing constraints, and their long-term investment and financing funds cannot be effectively supported [12], resulting in the frequent investment and financing strategies of "short-term debt and long-term use" of supply chain enterprises [13, 14]. It is not difficult to find that as a special investment and financing strategy, the short-term debt for long-term use not only has risk characteristics, but also has income characteristics.

On the one hand, according to the theory of agency cost hypothesis, the short-term debt for long-term use within a reasonable range helps to improve the regulatory responsibility of banks and other financial institutions as creditors of supply chain enterprises. Because the term of short-term debt is usually short, supply chain enterprises need to often sign credit contracts with creditors. Before each signing of short-term debt contracts, banks and other financial institutions need to reevaluate the operation risk and liquidity risk of supply chain enterprises to reduce the occurrence of bad debt losses. In addition, creditors of banks and other financial institutions can also supervise the aggressive and high-risk investment activities of corporate managers through the use of short-term loans [15, 16], thus reducing the principle-agency cost [17] and further reducing the moral hazard of senior managers and the operational risk of enterprises. On the other hand, according to the financing transaction cost theory, short-term debt for long-term use to a certain extent helps to improve debt contracts and reduce financing transaction costs of supply chain enterprises [18]. Kahl (2015) found that most American enterprises prefer to use short-term commercial paper to support long-term capital investment expenditure in the early stage of investment [5]. So as to reduce transaction costs and improve investment performance. Moreover, compared with long-term debt financing, short-term loans are easier to obtain approval [19], more conducive to improving the success rate of financing, and the interest rate is relatively lower [5]. However, with the continuous accumulation of short-term loans, when the dependence of supply chain enterprises on short-term loans exceeds the optimal level, debt problems such as excessive debt will also occur, which inevitably increases the possibility of default of supply chain enterprises [20, 21]. More importantly, short-term loans also have renewal risks [22]. When the project itself has high investment risk and low development prospect, its future profitability will be difficult to support the debt paying ability of the supply chain enterprise, and the capital turnover risk of the enterprise will gradually increase, thus expanding the loan renewal risk of the enterprise, making the enterprise fall into operating difficulties and financial crisis. Although companies can take measures such as rolling over debt and raising new debt to temporarily ease the crisis, they still face liquidity pressure. In other words, the excessive "short-term debt and long-term use" has imposed certain restrictions on the liquidity of supply chain enterprises’ funds, weakening the cash flexibility of supply chain enterprises. When there are large fluctuations and uncertainties in the macro-economy, this will inevitably affect the potential risk of default of supply chain enterprises.

Based on the above analysis, this paper believes that compared with the previous research results on the linear impact of short-term debt and long-term debt on corporate default risk [5, 23], considering the dual characteristics of short-term debt and long-term debt with both income effect and risk effect, although the analysis of the causes of the phenomenon of short-term loan and long-term investment helps to explain which methods can influence the behavior of enterprises’ short-term loan and long-term investment, it can not effectively clarify whether this radical term mismatch has a positive or negative effect on the long-term development of enterprises, and the academic research on this aspect is still blank. Therefore, it is necessary for us to further investigate the economic consequences of short-term loan and long-term investment, and clarify how it affects the long-term development of enterprises, so as to provide some reference for how to promote the stable development of the real economy.

In general, to a certain extent, the short-term debt for long-term use can give full play to the flexibility and low cost advantages of short-term debt and reduce the cost of financing transactions. However, driven by the motive of chasing profits, excessive use of short-term borrowing to support long-term project investment will increase the debt repayment pressure of supply chain enterprises, which will lead to business difficulties and liquidity risks of enterprises. It is not difficult to find that how the short-term debt for long-term use affects the default risk of enterprises depends on the level of long-term use of short-term debt. There is an obvious "double-edged sword" effect between the short-term debt for long-term use and the default risk of supply chain enterprises. There is not a simple linear relationship between the two, but there may be a U-shaped curve relationship. Therefore, this paper puts forward the following research hypotheses:

H1: There is a significant U-shaped relationship between the short-term debt for long-term use and the default risk of supply chain enterprises.

Qualitative change is an accumulation process of quantitative change, as is the behavior of short-term debt for long-term use of enterprises. Since the reform and opening up, under the background of the rapid expansion of fixed asset investment scale, China’s economy has ushered in a golden age of rapid growth. However, for a long time, compared with the rapidly developing economic environment, China’s financial system has been in the position of "financial repression," and the financing needs of enterprises cannot be met, resulting in the behavior of short-term debt for long-term use. To a certain extent, the short-term debt for long-term use is not only conducive to timely alleviating the financing pressure of enterprises, but also conducive to giving full play to the benefits of external supervision and standardizing the behavior of agents, so as to improve the scientific and effective decision-making of enterprise managers, improve the investment efficiency of enterprises, and have a positive impact on the performance of enterprises, so as to achieve the governance purpose of reducing the default risk of enterprises. In view of the fact that debt financing enterprises need to perform debt interest obligations on a fixed and regular basis, which reduces the management’s free control over cash flow to a certain extent, short-term debt for long-term use can reduce the agency cost between enterprise shareholders and enterprise managers, improve enterprise investment efficiency, strengthen enterprise performance, and then alleviate the default risk of enterprises.

When the short-term debt for long-term use exceeds the optimal level, debt problems such as excessive debt will also occur, and the short-term debt for long-term use will inevitably change qualitatively, which has a negative impact on the possibility of default of supply chain enterprises. Excessive short-term debt for long-term use will increase the liquidity risk of supply chain enterprises, reduce the productivity of enterprises [24] and investment efficiency, have an adverse inhibitory effect on the R&D investment of enterprises [25], damage the operating performance of enterprises [26, 27], and affect the credit loans and commercial credit rationing of supply chain enterprises, As a result, enterprises with excessive short-term debt for long-term use can only seek higher cost commercial loans, and ultimately increase the default risk of supply chain enterprises [28].

Based on this, this paper believes that the U-shaped relationship between the short-term debt for long-term use and the default risk of supply chain enterprises further affects the default risk of supply chain enterprises by affecting the performance of supply chain enterprises. Therefore, this paper puts forward the following research hypothesis:

H2: enterprise performance plays a mediating role in the process of short-term debt for long-term use affecting the default risk of supply chain enterprises.

**Fig 1:**
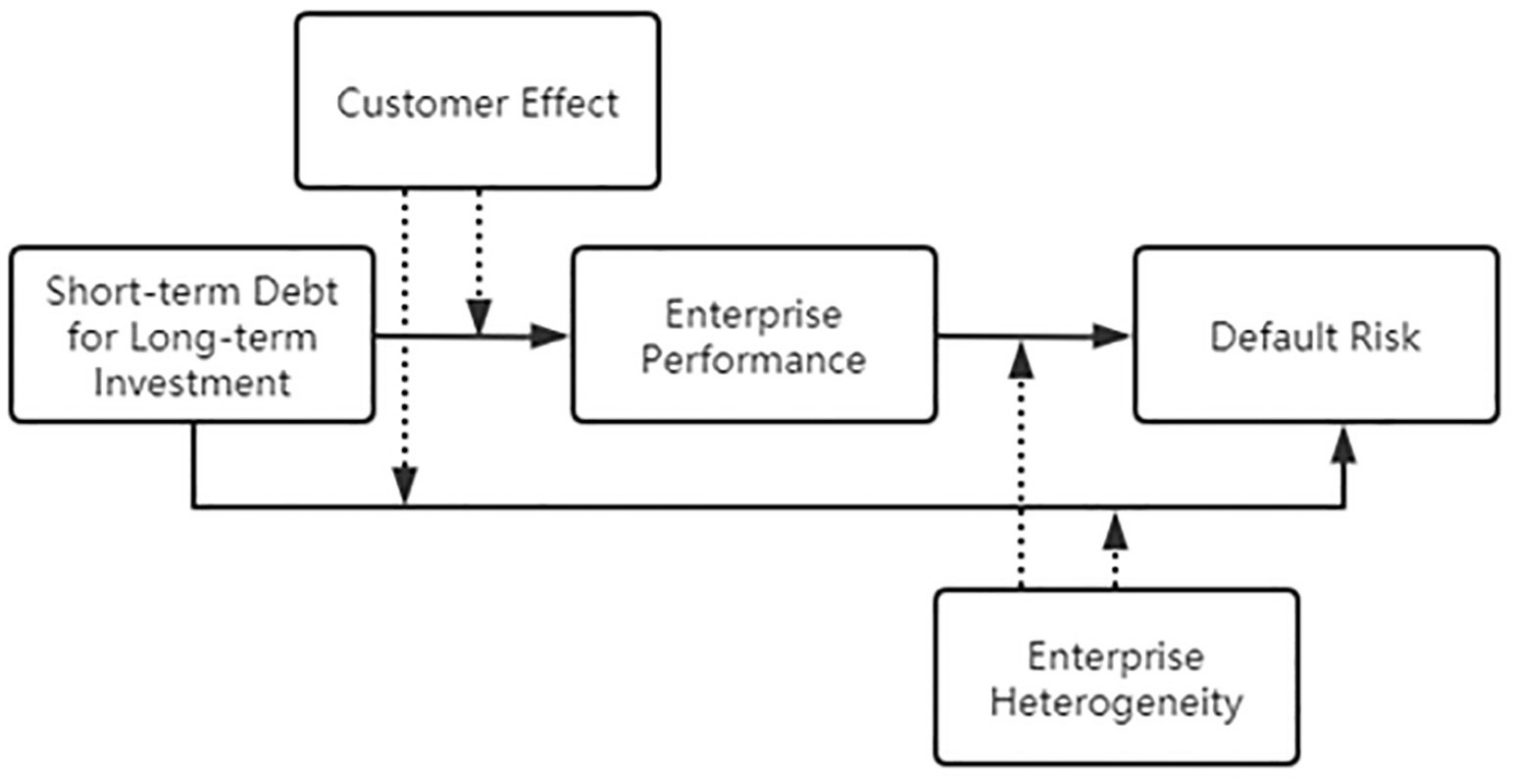
Flow chart of empirical model.

To sum up, the empirical model of this paper is shown in Fig 1.

## 3. Sample selection and research design

In 2007, China implemented the new accounting standards for Business enterprises. The research samples of this paper are China’s A-share listed enterprises from 2007 to 2021, and the financial data are from the CSMAR database, excluding the samples of financial listed enterprises. (All relevant data are within the manuscript and its Supporting Information files.)At the same time, in order to control the deviation caused by outliers on the estimation results, continuous variables are subjected to up and down 1% Winsorize processing. Finally, this paper obtains a total of 24,897 annual samples of 3847 listed enterprises from 2007 to 2021.

In order to explore the impact of " short-term debt for long-term use" on the default risk of supply chain enterprises, and study whether enterprise performance plays an intermediary role in the impact of short-term debt for long-term use on the default risk of supply chain enterprises, this paper believes that the first priority should be to verify the existence of the phenomenon of " short-term debt for long-term use" in China’s capital market. Therefore, Based on the research of McLean and Zhao (2014), we construct our "investment short-term loan" sensitivity model using the "investment cash flow" sensitivity model to test the dependence of supply chain enterprises on short-term debt. The sensitivity model constructed is as follows:

Model 1:

$$INV_{it}=\alpha_{0}+\alpha_{1}CFO_{it}+\alpha_{2}LongDebt_{it}+\alpha_{3}ShortDebt_{it}+\alpha_{4}Control_{it}+\Sigma Industry+\Sigma Year+\varepsilon_{it}$$ (1)

Secondly, in order to analyze the impact of short-term debt for long-term use on the default risk of supply chain enterprises, we constructs the empirical model (2) of this paper for analysis.

Model 2:

$$EDF_{it}=\beta_{0}+\beta_{1}SFLI_{it}+\beta_{2}SFLI^{2}{}_{it}+\beta_{3}Control_{it}+\Sigma Industry+\Sigma Year+\varepsilon_{it}$$ (2)

Finally, in order to test the Mesomeric effect of enterprise performance in the process of long-term use of short-term debt on the default risk of supply chain enterprises, we introduced the U-shaped Mesomeric effect test method, and built the transmission mechanism measurement model (3) and model (4) based on the empirical model (2) Model 3:

$$TQ_{it}=\eta_{0}+\eta_{1}SFLI_{it}+\eta_{2}SFLI^{2}{}_{it}+\eta_{3}Control_{it}+\Sigma Industry+\Sigma Year+\varepsilon_{it}$$

Model 4:

$$EDF_{it}=\lambda_{0}+\lambda_{1}TQ_{it}+\lambda_{2}SFLI_{it}+\lambda_{3}SFLI^{2}{}_{it}+\lambda_{4}Control_{it}+\Sigma Industry+\Sigma Year+\varepsilon_{it}$$

The explained variables and explanatory variables of Models (1) to (4) are as follows:

Explained variables: investment "INV", default probability "EDF" and enterprise performance "TQ":

In Model (1), the explained variable investment "INV" comes from the annual cash flow statement of supply chain enterprises—"cash paid for the purchase and construction of fixed assets, intangible assets and other long-term assets," and eliminates the scale effect through the total assets of the previous year;

In Model (2), the explained variable default probability "EDF" is calculated by the KMV model. KMV model is a major reform of the traditional default rate measurement method. This paper uses KMV model as the basis to measure the default probability of supply chain node enterprises, and its advantages are as follows: First of all, compared with the historical data based on the previous book information of enterprises, the KMV model selects the real-time data based on the stock market, which not only makes the model data more forward-looking, but also can better reflect the current debt default situation of the supply chain node enterprises, so as to better predict the default probability of enterprises. Secondly, KMV model is based on the "structural model" of modern corporate finance and option theory, which is more convincing in predicting the default probability of enterprises to a certain extent. Finally, the KMV model uses the publicly disclosed financial data of node enterprises to estimate the default probability, which makes the model more suitable for listed enterprises.

The KMV model regards the book value of the debt of the node enterprise as the strike price of a European call option. When the book value of the debt of the node enterprise is higher than the total market value of the enterprise’s assets, the enterprise has the risk of default. The asset market value and asset market value volatility of node enterprises can be solved from the following simultaneous equations, and the specific calculation is as follows:

$$\left\{{}_{\sigma_{E}=\left[VN(d_{1})\right]\sigma_{V}/E}^{E=VN(d_{1})-De^{-rt}N(d_{2})}\right.$$

In this equations,

$$d_{1}=\left[ln(V/D+(r+\sigma_{V}^{2}/2\right]T/\sigma_{V}\sqrt{T}$$

$$d_{2}=d_{1}-\sigma_{V}\sqrt{T}$$

Market value of enterprise stock"E" = (number of outstanding shares × price of outstanding shares + number of non-outstanding shares × price of non-outstanding shares). "σE" is the annual standard deviation of stock return rate, "V" is the market value of enterprise assets, and the book value of enterprise debt"D" = current liabilities +0.5× long-term liabilities. "T" is the debt maturity, "σV" is the market value volatility of firm assets, and "r" is the risk-free interest rate. Calculate the expected default probability "EDF" of the enterprise at time "T":

$$\begin{array}{ll} EDF & =P(V_{T}\leq D)=P\left\{V_{0}exp\left[\left(\mu -\sigma_{V}^{2}/2\right)T+\sigma_{V}\sqrt{T}\varepsilon \right]\leq D\right\}\\ & =P\left[-\frac{ln(V_{0}/D)+\left(\mu -\sigma_{V}^{2}/2\right)T}{\sigma_{V}\sqrt{T}}\geq \varepsilon \right]\\ & =N\left[-\frac{ln(V_{0}/D)+\left(\mu -\sigma_{V}^{2}/2\right)T}{\sigma_{V}\sqrt{T}}\right]=N\left[-DD\right] \end{array}$$

In Formula (20), the distance between the value of enterprise assets and the value of debt is defined as the default distance"DD"

$$DD=-\frac{ln(V_{0}/D)+\left(\mu -\sigma_{V}^{2}/2\right)T}{\sigma_{V}\sqrt{T}}$$

Obviously, "N" is the cumulative standard normal distribution function. The larger the calculated "EDF"value is, the higher the default risk of the enterprise. On the contrary, the smaller the E value, the lower the default risk of the enterprise.

In Model (3), enterprise performance "TQ" is the explained variable, and Tobin’s Q value is selected to measure enterprise performance with reference to most literature practices.

Explanatory variables: net cash flow "CFO", long-term credit increment "Long-Debt", short-term credit increment "Short-Debt", short-term debt for long-term use "SFLI":

In model (1), the explanatory variable net cash flow "CFO"comes from the annual cash flow statement of supply chain enterprises—"net cash flow from operating activities", and the scale effect is eliminated through the total assets of the previous year; The explanatory variables long-term credit increment "Long-Debt", short-term credit increment"Short-Debt", which represent the new short-term and long-term credit volume of the supply chain enterprise in the current period, are calculated and solved by using the annual balance sheet and annual cash flow statement data of the supply chain enterprise, and the scale effect is eliminated by the total assets of the previous year; The definition of other control variables will be described in detail below (see Table 1 for details).

**Table 1:** Relevant variables and definitions.

*Symbol*	*Name*	*Definition*
*INV*	*Investment Amount*	Cash paid for the purchase and construction of fixed assets, intangible assets and other long-term assets; Take its logarithm
*EDF*	*Default Risk*	The default probability is measured by KMV model
*TQ*	*Enterprise Performance*	Tobin Q value
*CFO*	*Net Cash Flow*	Net cash flow from operating activities
*LongDebt*	*Long term Credit Increment*	Current long-term credit increment = current long-term borrowings+non current liabilities due within one year—previous long-term borrowings; Take its logarithm
*ShortDebt*	*Short term Credit Increment*	Current short-term credit increment = cash received from borrowings—current increase in long-term borrowings; Take its logarithm
*SFLI*	*short-term* *debt for long-term* *use*	Short term debt and long-term use = Cash Expenditure for investment activities such as the purchase and construction of fixed assets—(current increase in long-term borrowings+current increase in equity+net cash flow from operating activities+cash inflow from the sale of fixed assets)
*LEV*	*Capital Structure*	Total liabilities of the current period/total assets of the current period
*STATE*	*Nature of Property Rights*	1 for state-owned enterprises and 0 for non-state-owned enterprises
*SIZE*	*Enterprise Size*	Natural logarithm of total assets
*ROA*	*Return on Assets*	Net profit/average balance of total assets
*AGE*	*Enterprise Age*	Years of listed companies
*INDUSTRY*	*Industry*	Industry dummy variables are used to distinguish the industries in which enterprises are located according to the 2012 industry classification standards of the China Securities Regulatory Commission
*YEAR*	*Annual Control*	Annual dummy variable

In model (2), the explanatory variables of short-term debt for long-term use "SFLI", "SFLI"and"SFLI2"are the linear term and quadratic term of short-term debt for long-term use of supply chain enterprises respectively, which are calculated and solved by using the data of annual balance sheet and annual cash flow statement of supply chain enterprises, and the scale effect is eliminated by the total assets of the previous year; Referring to the method proposed by Zhong Kai et al. (2016) and Liu xiaoguang and Liu yuanchun (2019) [23, 29], this paper depicts the measurement index of "short-term debt for long-term use", and uses the measurement method of Zhong Kai et al. For reference, calculates the method of "short-term debt for long-term use" to define proxy variables, and takes the measurement method of Liu xiaoguang and Liu yuanchun(2019) as the robustness test index of this paper. The relevant definitions of other control variables and specific variables are shown in Table 1.

## 4. Empirical analysis

### 4.1 Descriptive statistics

According to the descriptive statistical results in Table 2, first of all, from the statistical results of investment and credit amount, it is not difficult to find that China’s supply chain enterprises do have a serious phenomenon of short-term debt for long-term use, and the maximum value of short-term debt for long-term use is 0.456, the minimum value is -5.108, and the standard deviation is 0.303, indicating that there are obvious differences in the short-term debt for long-term use behavior of supply chain enterprises. To some extent, it shows that the long-term credit funds obtained by Chinese listed companies are significantly lower than short-term credit funds; Secondly, the average default risk of supply chain enterprises is about 0.177. Combined with the sorted data, especially in the period of economic fluctuations (such as the subprime mortgage crisis in 2008, the new crown impact in 2020 and other nodes), the default risk fluctuated greatly, and the maximum value reached an amazing 0.955. It can be seen that under the dual influence of its own micro factors and macro fluctuations, enterprises have very serious potential default risks, It is worthy of academic attention; In addition, the average Tobin Q value of enterprise performance is 2.094, the standard deviation is 3.157, and the maximum value is 259.1, indicating that there are obvious differences in the performance (return on investment) of data enterprises; Finally, the standard deviations of the remaining variables such as financial leverage (LEV), return on equity (ROA), enterprise size (SIZE), enterprise age (AGE), and property right nature (STATE) are 1.114, 0.325, 1.352, 6.076, and 0.482, respectively. The difference between the value of the control variables and the average level is low, indicating that the variables have good stability.

**Table 2:** Descriptive statistics of main variables.

*variables*	*mean*	*SD*	*Min*	*Max*
*INV*	18.458	1.934	7.346	26.525
*KMV*	0.177	0.109	0	0.955
*CFO*	0.045	0.088	-4.270	2.222
*LongDebt*	18.767	2.658	-20.101	25.615
*ShortDebt*	19.944	2.103	-20.101	27.763
*SFLI*	-0.108	0.303	-5.108	0.456
*LEV*	0.460	1.114	0.007	178.300
*TQ*	2.094	3.157	0	259.100
*STATE*	0.368	0.482	0	1
*ROA*	0.027	0.325	-30.960	10.400
*SIZE*	22.060	1.352	15.580	28.480
*AGE*	17.070	6.076	0	62.000

### 4.2 Sensitivity analysis of short-term debt and long-term use

In order to explore the impact of "short-term debt for long-term use" on the default risk of supply chain enterprises and study the intermediary effect of financing constraints, this paper first verifies the existence of the radical financing phenomenon of "short-term debt for long-term use" in China’s capital market. This study refers to the analysis idea of "investment cash flow", and uses the model (1) to conduct a full sample regression on the sensitivity test of "investment short-term loans", which provides the necessary objective confirmation and theoretical basis for this study. Table 3 reports the sensitivity analysis results of short-term debt for long-term use. The preliminary study found that the sensitivity analysis of "investment short-term loan" supports the view of this study, showing a significant positive correlation, which indicates that there is indeed "short-term debt for long-term use" behavior in China’s supply chain enterprises. Enterprise investment depends not only on investment opportunities, but also on various financing sources. High growth companies will not easily adopt the radical financing method of "short-term loan and long-term investment";

**Table 3:** Sensitivity analysis of short-term debt and long-term use.

	(1)		(1)
	*INV*		*INV*
*CFO*	1.055***	*AGE*	-0.0461***
	(8.52)		(-13.77)
*CQDK*	0.0472***	*ROA*	0.00655**
	(9.89)		(3.03)
*DQDK*	0.0312***	*_cons*	1.203**
	(3.95)		(2.61)
*LEV*	-0.959***	*Industry*	Yes
	(-15.38)		
		*Year*	Yes
*M2*	-0.256***	*N*	24897
	(-7.53)	*R* ^*2*^	0.5105
*SIZE*	0.943***		
	(57.30)		

*t* statistics in parentheses

* *p* < 0.05,

** *p* < 0.01,

*** *p* < 0.001

### 4.3 Short-term debt for long-term use and default risk of supply chain enterprises

**Fig 2:**
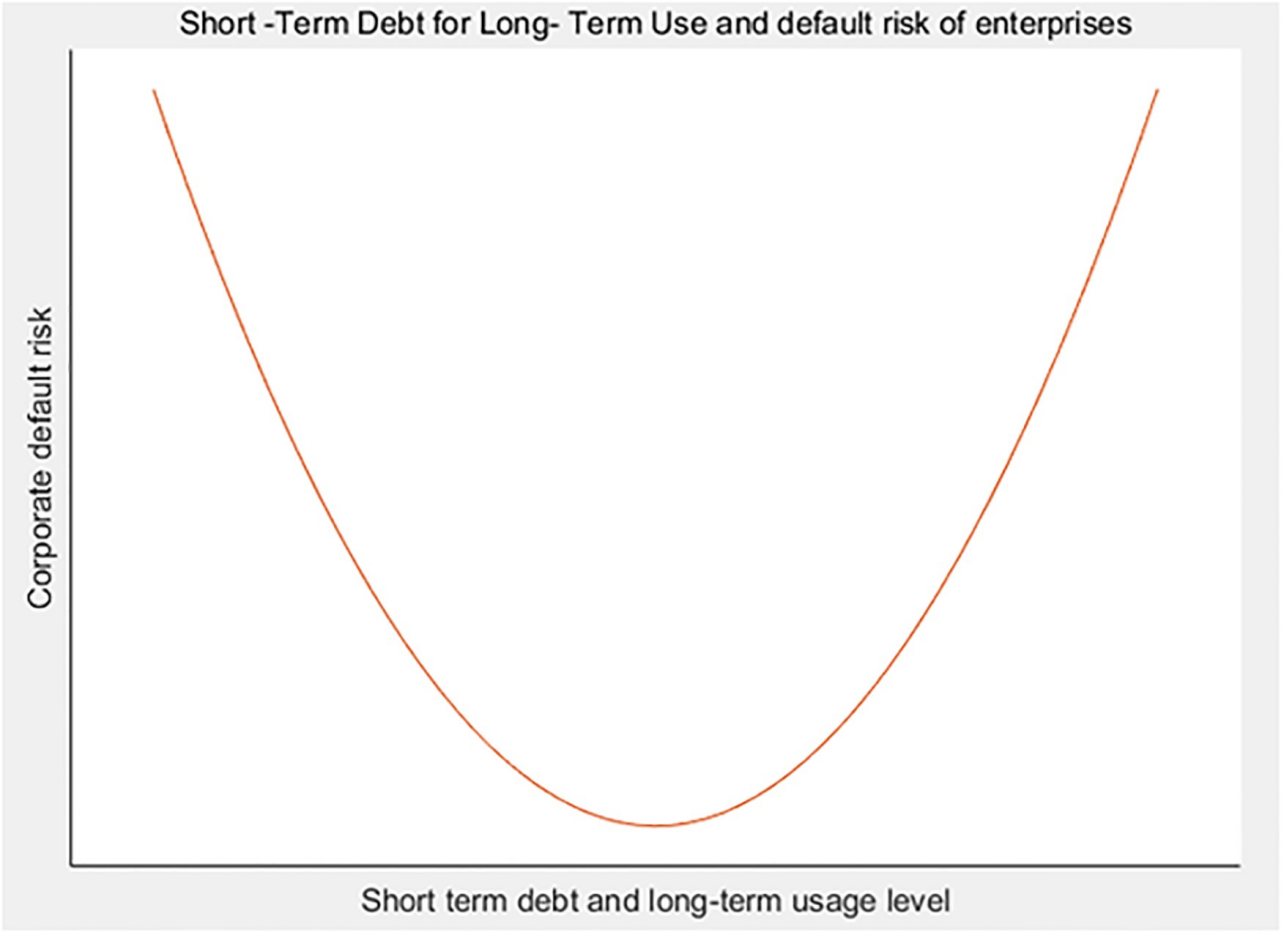
Short-term debt for long-term use and default risk of supply chain enterprises.

(2) to (4) of Table 4 show the correlation regression analysis results of short-term debt for long-term use and default risk of supply chain enterprises. According to the regression results in column (2) of Table 4, first of all, without introducing other relevant control variables, there is a significant correlation between the short-term debt for long-term use and the default risk of supply chain enterprises, and the linear term coefficient of 0.0266 for short-term debt for long-term use is significantly negative at the level of 1%; The quadratic term coefficient of 0.00697 is significantly positive at the 1% level, indicating that the relationship between the short-term debt for long-term use and the default risk of supply chain enterprises is not a simple linear relationship, but a U-shaped relationship. Secondly, according to the regression results in column (4) of Table 4, after introducing the relevant control variables, there is still a significant correlation between the short-term debt for long-term use and the default risk of supply chain enterprises, and the linear term coefficient of 0.0241 for short-term debt for long-term use is significantly negative at the level of 1%; The quadratic term coefficient of 0.00544 is significantly positive at 1% level. The short-term debt for long-term use still has a significant U-shaped relationship with the default risk of enterprises: on the left side of the U-shaped relationship, the short-term debt for long-term use has a negative correlation with the default risk of supply chain enterprises; On the right side of the U-shape, there is a positive correlation between the short-term debt for long-term use and the default risk of supply chain enterprises (see Fig 2). This research result shows that whether the use of short-term debt for a long time is too high or too low, it will have an adverse impact on the default risk of supply chain enterprises, so H1 hypothesis in this paper is true. It shows that short-term loans have a certain governance effect, can inhibit the moral hazard of enterprise management participating in high-risk investment projects, and help reduce enterprise risk. However, with the increasing degree of short-term debt and long-term use, enterprises are more likely to face the test of project investment payback period, which is more likely to lead to difficulties in capital turnover, and have to "borrow the new to repay the old" at a higher interest cost, The new debt financing is more used to meet the needs of enterprise financial operation than actual business investment and R&D innovation. It is likely to enter the dilemma of "borrowing new to repay old" → "borrowing new to repay interest" → "balance sheet deterioration", thereby worsening enterprise performance and debt risk. At the same time, it is worth noting that the relevant control variables such as cash flow, return on assets and other indicators are significantly negatively correlated with the default risk of supply chain enterprises, which also explains the importance of return and cash flow to the default risk of supply chain enterprises. Especially in recent years, affected by the epidemic and Sino US trade friction, most of the important reasons for the default of supply chain enterprises are due to the shortage of cash flow.

**Table 4:** Long term use of short-term debt and default risk of supply chain enterprises.

	(2)	(3)	(4)
	*KMV*	*KMV*	*KMV*
*SFLI*	-0.0266***		-0.0250***
	(-12.39)		(-11.63)
*SFLI* ^*2*^	0.00697***		0.00598***
	(9.55)		(8.40)
*CFO*		-0.108***	-0.114***
		(-15.48)	(-15.17)
*LEV*		-0.00150**	0.00101
		(-2.66)	(1.85)
*SIZE*		0.0175***	0.0153***
		(36.11)	(30.95)
*AGE*		0.000172	0.000641***
		(1.54)	(5.35)
*ROA*		-0.000838***	-0.00104***
		(-4.20)	(-5.30)
*M2*		0.0173***	-0.0286***
		(10.70)	(-14.57)
*_cons*	0.177***	-0.457***	0.237***
	(221.84)	(-19.35)	(8.38)
*Industry*	Yes	Yes	Yes
*Year*	Yes	Yes	Yes
*N*	24897	24897	24897
*R* ^*2*^	0.0029	0.1561	0.2237

*t* statistics in parentheses

* *p* < 0.05,

** *p* < 0.01,

*** *p* < 0.001

### 4.4 Short-term debt for long-term use, enterprise performance and default risk of supply chain enterprises

Table 5 (5) to (7) lists the mediating effect of enterprise performance on the default risk of supply chain enterprises in short-term debt for long-term use. It is worth noting that in order to exclude the highly correlated effect between the enterprise capital structure and other financing constraint indicators in this paper, this paper eliminated the influencing factor of enterprise capital structure in the analysis of intermediary effect, so as to ensure the reliability of the research results more accurately. Among them, the regression analysis in column (5) of Table 5 reported that the linear term coefficient of 0.025 for short-term debt and long-term debt was significantly negative at the level of 1%; The quadratic term coefficient of 0.00598 is significantly positive at the 1% level, which supports the significance analysis of β_1_ and β_2_ in the research model (2). The regression results in column (6) of Table 5 show that the linear term coefficient of 0.187 for short-term debt for long-term use is significantly negative at the 1% level; The quadratic term coefficient of 0.0945 is significantly positive at the 1% level, which supports the significance analysis of η_1_ and η_2_ in the model (3) of this study. The regression results in column (7) of Table 5 show that the effect of enterprise performance on the default risk of supply chain enterprises is significantly positive at the level of 1% (the influence coefficient is 0.00242), indicating that enterprise performance plays a partial intermediary role in the effect of short-term debt for long-term use on the default risk of supply chain enterprises, and also supports the significance analysis of λ_1_ and λ_2_ in the research model (4). This shows that the long-term use of short-term debt may affect the overall performance of enterprises by increasing the risk compensation premium for stakeholders or aggravating the distortion of enterprise innovation incentives, and then affect the default risk of enterprises. To sum up, the research results of this paper verify the transmission path of short-term debt for long-term use → enterprise performance → supply chain enterprise default risk, and the research hypothesis of this paper is H2.

**Table 5:** Short-term debt for long-term use, enterprise performance and default risk of supply chain enterprises.

	(5)	(6)	(7)
	*KMV*	*TQ*	*KMV*
*SFLI*	-0.0250***	-0.187***	-0.0241***
	(-11.63)	(-3.31)	(-11.21)
*SFLI* ^*2*^	0.00598***	0.0945***	0.00544***
	(8.40)	(5.06)	(7.64)
*TQ*			0.00242***
			(11.12)
*CFO*	-0.114***	-1.487***	-0.111***
	(-15.17)	(-7.01)	(-14.74)
*LEV*	0.00101	0.551***	-0.000403
	(1.85)	(38.17)	(-0.72)
*SIZE*	0.0153***	-0.811***	0.0170***
	(30.95)	(-40.88)	(32.93)
*AGE*	0.000641***	0.0512***	0.000558***
	(5.35)	(10.19)	(4.65)
*ROA*	-0.00104***	-0.0207***	-0.00103***
	(-5.30)	(-4.06)	(-5.27)
*M2*	-0.0286***	-0.102	-0.0283***
	(-14.57)	(-1.62)	(-14.45)
*_cons*	0.237***	20.39***	0.192***
	(8.38)	(23.09)	(6.74)
*Industry*	Yes	Yes	Yes
*Year*	Yes	Yes	Yes
*N*	24897	24897	24897
*R* ^*2*^	0.2237	0.1032	0.2130

*t* statistics in parentheses

* *p* < 0.05,

** *p* < 0.01,

*** *p* < 0.001

## 5. Further analysis and robustness test

### 5.1 Customer effect analysis

As the core element of supply chain enterprises, customer effect (customer concentration) not only affects the investment, financing and the Cost Stickiness of supply chain enterprises, but also affects the short-term debt for long-term use utilization level and default risk of supply chain enterprises. On the one hand, the theory of "value co creation" holds that positive customer effect will help strengthen the positive development of supply chain cooperation and promote the value creation of supply chain. The highly concentrated customer effect has contributed to the main performance income of supply chain enterprises. The more high-quality customers with high consumption, the more considerable the operating income of enterprises. The enhancement of profitability establishes reliable protection barriers for the performance of supply chain enterprises and market changes, and further releases positive signals of high value to the capital market and banking system, so as to improve the financing ability of supply chain enterprises, optimize the debt financing structure and term limit of supply chain enterprises, and make supply chain enterprises more flexible in controlling the level of short-term debt for long-term use, And then affect the default risk of enterprises to a certain extent. On the other hand, according to the theory of "value plunder", the higher the customer concentration of a supply chain enterprise, the higher the degree of exclusive payment and dependence of the enterprise on its main cooperative customers, and the vulnerability of the enterprise will also increase. Once the customer relationship breaks down and customers have serious business risks, the negative impact of the customer effect will easily rely on the intermediary carriers such as material flow, information flow and financial flow in the supply chain network, It will cause huge losses to the performance of supply chain enterprises. As a result, financial institutions such as banks will also more strictly restrict the financing loans of such supply chain enterprises, such as setting higher loan interest thresholds and financing loan terms (wangjunqiu et al., 2015 [30]). To a large extent, this also affects the level of short-term debt and long-term debt and default risk of supply chain enterprises.

**Fig 3:**
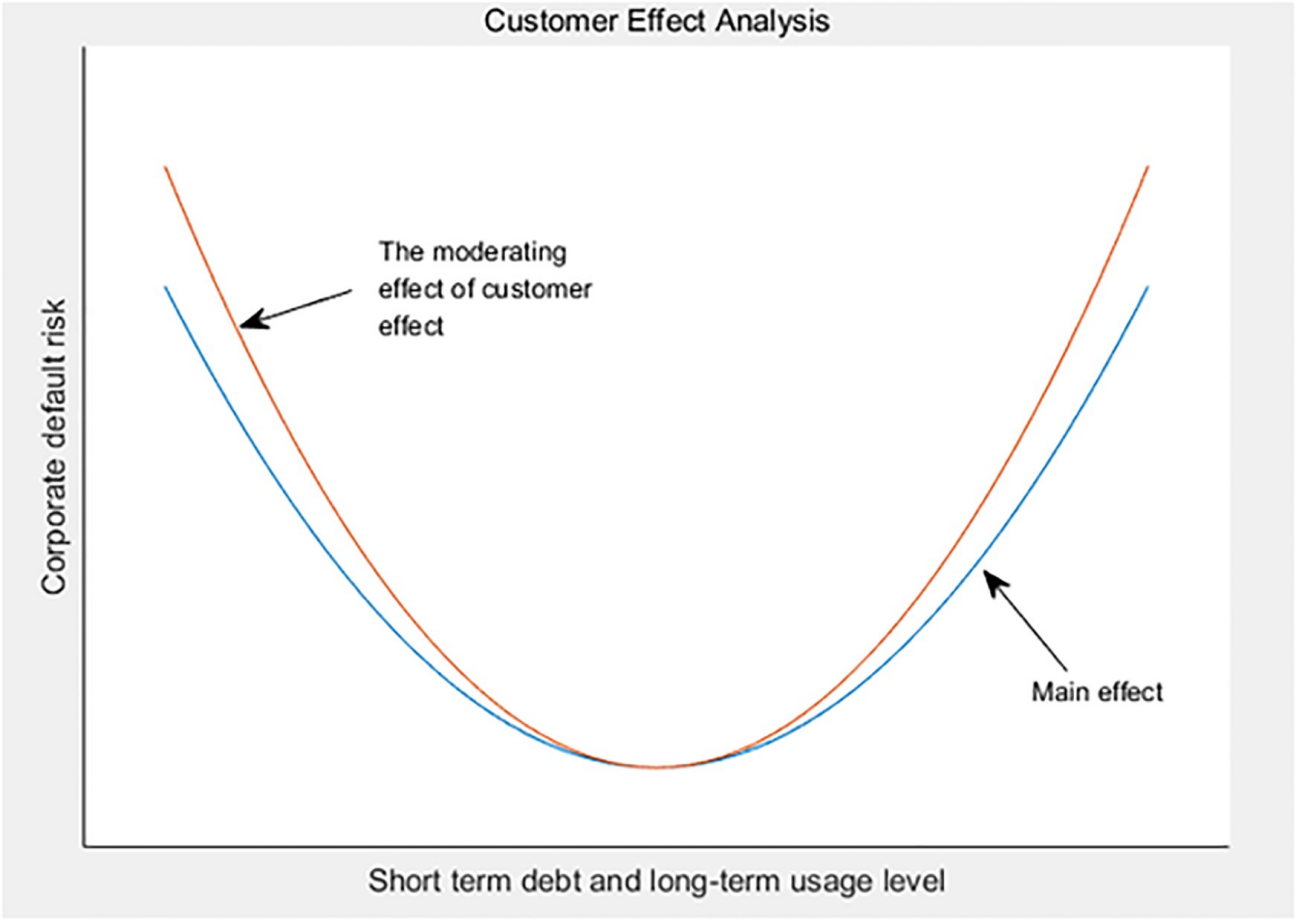
Customer effect analysis.

Therefore, on the basis of the original research, we take supply chain customer concentration (buy) as the proxy variable of customer effect, and introduce the moderating effect of customer effect and the linear term and quadratic term interactive terms of short-term debt for long-term use to further investigate whether the relationship between short-term debt for long-term use and supply chain enterprise default risk is affected by customer effect. Table 6 lists the moderating effect of customer effect. The test results show that customer effect × The square of short-term debt for long-term use has a significant positive impact on the default risk of supply chain enterprises (interaction coefficient 0.0000787, p<0.05). This shows that the U-shaped relationship between short-term debt for long-term use on the default risk of supply chain enterprises is positively regulated by the customer effect, that is, when the customer concentration is at a high level, the U-shaped relationship between short-term debt for long-term use on the default risk of supply chain enterprises will be strengthened (see Fig 3).

**Table 6:** Customer effect analysis test.

	(8)	(9)
	*KMV*	*KMV*
*SFLI*	-0.0248***	-0.0303***
	(-11.51)	(-8.18)
*SFLI* ^*2*^	0.00600***	0.00549***
	(8.44)	(7.71)
*BUY*	0.0000771**	0.0000782*
	(2.59)	(2.46)
BUY*SFLI		0.0000147
		(0.13)
BUY*SFLI ^*2*^		0.0000787**
		(2.69)
*Controls*	Yes	Yes
*_cons*	0.243***	0.202***
	(8.49)	(7.00)
*Industry*	Yes	Yes
*Year*	Yes	Yes
*N*	24897	24897
*R* ^*2*^	0.221	0.220

*t* statistics in parentheses

* *p* < 0.05,

** *p* < 0.01,

*** *p* < 0.001

### 5.2 Analysis of enterprise heterogeneity

**Fig 4:**
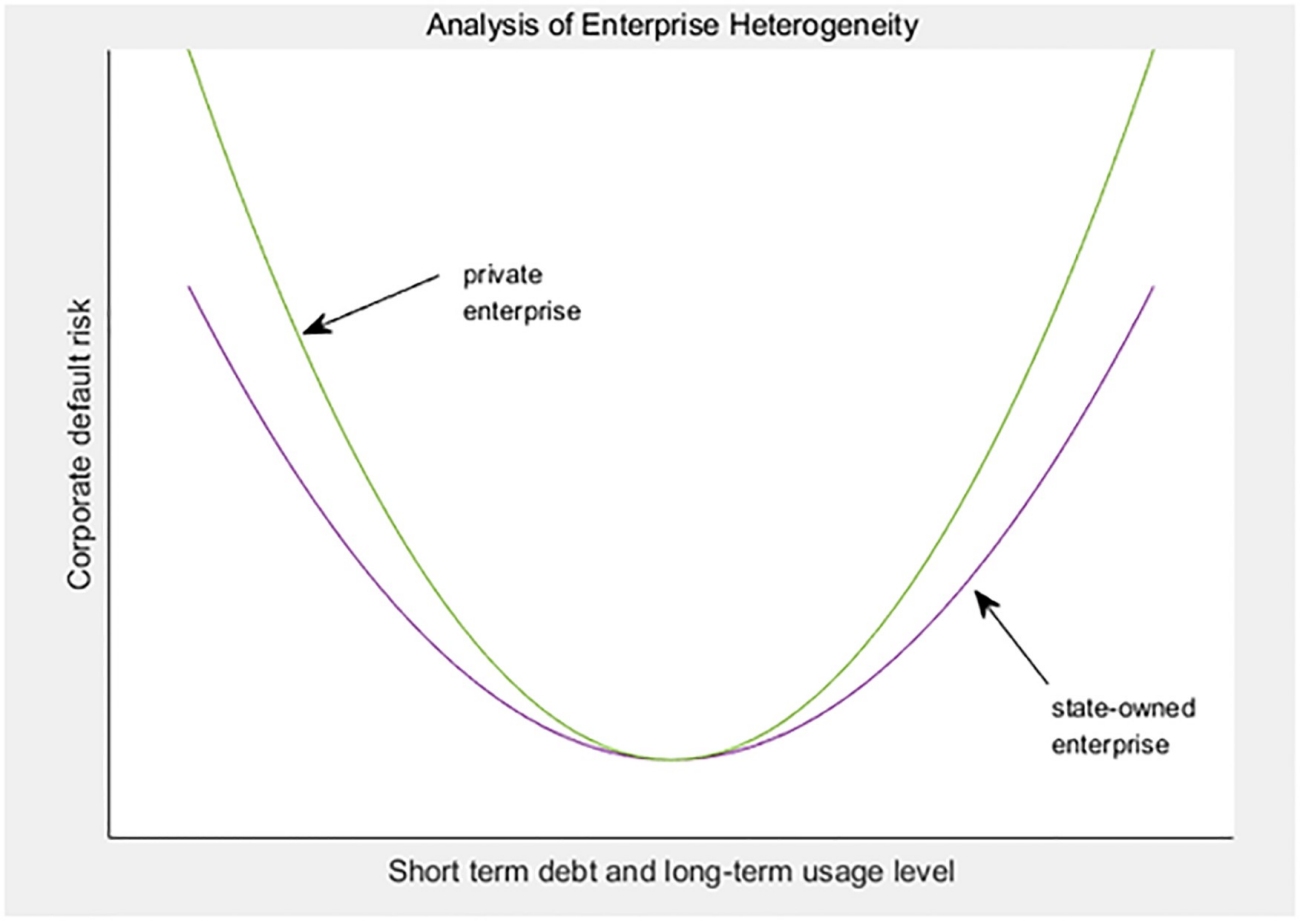
Enterprise heterogeneity analysis.

The difference in the nature of property rights is the most significant manifestation of enterprise heterogeneity. It is undeniable that state-owned enterprises are far superior to private enterprises in terms of resources, reputation and market position, no matter from the perspective of background or financing ability. It is precisely based on these advantages that state-owned enterprises often have higher bargaining chips in negotiations. It is worth noting that due to the relationship between government and enterprises, state-owned enterprises are more conducive to the implicit guarantee of the government, so they have more competitive advantages in the market. Under the influence of macroeconomic fluctuations, due to the natural enjoyment of some special local preferential policies and the endorsement and guarantee of local government departments, it is easier for state-owned enterprises to obtain the favor and support of core enterprises to a large extent; In addition, compared with private enterprises, the governance system of state-owned enterprises is generally more sound, the property right structure is also more clear, which is more helpful to make up for the negative impact caused by information asymmetry, and help them have a better position in dealing with the problem of credit risk contagion in the supply chain. Therefore, based on the research results and the heterogeneity of enterprises, this paper makes a regression test. First, in order to determine whether the short-term debt for long-term use by private enterprises stems from "financial discrimination", this paper conducts a sensitivity analysis on the "investment short-term loans" of state-owned and private enterprises in batches by dividing the nature of property rights. Finally, it tests the differential effect of short-term debt for long-term use on supply chain enterprises under different property rights, The main conclusions have not changed substantially. See Table 7 and Fig 4 below for specific empirical results.

**Table 7:** Enterprise heterogeneity analysis.

	(10)	(11)	(12)	(13)
	*INV*	*INV*	*KMV*	*KMV*
	*state-owned enterprise*	*private enterprise*	*state-owned enterprise*	*private enterprise*
*CFO*	0.793***	1.189***	-0.0602***	-0.137***
	(4.15)	(7.35)	(-5.05)	(-13.88)
*CQDK*	0.0537***	0.0423***		
	(7.06)	(6.94)		
*DQDK*	0.0295**	0.0318**		
	(2.62)	(2.89)		
*SFLI*			-0.0176***	-0.0289***
			(-4.09)	(-10.96)
*SFLI* ^*2*^			0.00553***	0.00513***
			(3.95)	(6.16)
*Controls*	Yes	Yes	Yes	Yes
*_cons*	3.499***	0.236	0.328***	0.111**
	(4.68)	(0.38)	(7.54)	(2.80)
*Industry*	Yes	Yes	Yes	Yes
*Year*	Yes	Yes	Yes	Yes
*N*	10877	14020	10877	14020
*R* ^*2*^	0.5707	0.4257	0.1847	0.2176

*t* statistics in parentheses

* *p* < 0.05,

** *p* < 0.01,

*** *p* < 0.001

### 5.3 Robustness test

In order to test the robustness of the U-shaped relationship between the short-term debt for long-term use and the default risk of supply chain enterprises, this paper makes further tests in the following aspects: (1) fixed effect test (FE). So as to avoid the relevant default risk of supply chain enterprises being interfered by factors such as enterprise cultural background, local economic regulations and policies, financial policies and so on, and then affect the accuracy of the study, this paper conducts a fixed effect test on panel data, After inspection, the main conclusions have not changed substantially, and the specific empirical results are listed in column (14) of Table 6; (2) To reconstruct the variable test (CV), this paper refers to the practice of Liu xiaoguang (2019) to measure the long-term use of short-term debt of supply chain enterprises by constructing the ratio difference between short-term debt and short-term assets (primary term SFLI (1) and secondary term SFLI (1) 2). The indicator believes that the smaller the difference between the ratio of short-term liabilities and short-term assets of an enterprise, the lower the level of short-term debt for long-term use. After inspection, the main conclusions have not changed substantially. See (15) in Table 6 below for specific empirical results; (3) The lag period test (LT1), in order to avoid the reverse causality of this research conclusion, that is, the supply chain enterprises’ reduction or excessive short-term debt and long-term use behavior is caused by the high default risk of supply chain enterprises. This paper conducted a lag period test on the core variable (short-term debt for long-term use). After the test, the main conclusion has not changed substantially. See (16) in Table 8 below for the specific empirical results.

**Table 8:** Robustness test.

	(14)	(15)	(16)
	*FE*	*CV*	*LT1*
*SFLI*	-0.0236***		-0.783**
	(-10.22)		(-2.76)
*SFLI* ^*2*^	0.00536***		1.172***
	(7.07)		(12.58)
*SFLI(1)*		-0.0103*	
		(-2.54)	
*SFLI(1)* ^*2*^		0.0152*	
		(2.17)	
*Controls*	Yes	Yes	Yes
*_cons*	0.0483	0.200***	-6.069
	(0.40)	(6.89)	(-1.23)
*Industry*	Yes	Yes	Yes
*Year*	Yes	Yes	Yes
*N*	24897	24897	20711
*R* ^*2*^	0.1805	0.2142	0.4887

*t* statistics in parentheses

* *p* < 0.05,

** *p* < 0.01,

*** *p* < 0.001

## 6. Conclusion and enlightenment

Based on the research sample of Shanghai and Shenzhen A-share listed enterprises from 2007 to 2021, this paper objectively verifies the existence of "short-term debt for long-term use" in China’s supply chain enterprises by constructing the sensitivity model of "investment short-term loan", empirically tests the impact of short-term debt for long-term use on the default risk behavior of supply chain enterprises, and further examines the mediating effect and regulatory effect between the two. It is found that there is a significant U-shaped curve relationship between the short-term debt for long-term use and the default risk of supply chain enterprises, rather than a simple linear relationship. To a certain extent, the short-term debt for long-term use can give full play to the flexibility and low-cost advantages of short-term debt, reduce the cost of financing transactions, and thus help reduce the risk of default; However, the excessive use of short-term loans to support long-term project investment will increase the debt repayment pressure of supply chain enterprises, further lead to business difficulties and liquidity risks, which will worsen the default risk of enterprises. In addition, this study also deduces the mediating effect of further study on the impact of short-term debt and long-term debt on the default risk of supply chain enterprises, and finds that there is an intermediary transmission path between the two, namely, short-term debt for long-term use → enterprise performance → supply chain enterprise default risk. Finally, customer effect and enterprise heterogeneity play a moderating role in the impact of short-term debt and long-term debt on the default risk of supply chain enterprises.

Since the outbreak of the financial crisis and the new crown epidemic, the long-term "financial repression" environment has made the long-term investment and financing funds of most small and medium-sized enterprises in China’s supply chain unable to be effectively supported. Although a certain degree of short-term debt for long-term use can provide liquidity support for the investment of supply chain enterprises and alleviate the financing constraints of supply chain enterprises, the excessive level of "short-term debt for long-term use" is easy to amplify the short-term debt repayment pressure of enterprises, trigger the liquidity risk and operation risk of supply chain enterprises, and finally aggravate the default risk of enterprises. Based on this, this paper attempts to give some suggestions: first, we should strengthen the risk awareness of enterprises, establish the risk oriented concept of enterprises, and at the same time, we should be alert to the harm of potential risks and strengthen the monitoring of enterprise risks while pursuing enterprise profits; Second, we should re-examine the function of short-term debt for long-term use, make overall planning for enterprise investment, recognize the dual characteristics of short-term debt and long-term use of income effect and risk effect, and build the corresponding risk prediction system based on the enterprise’s own development factors; Third, the government and relevant financial institutions should work together to improve the relevant systems and structures of the capital market, strengthen the standardized examination and approval management of long-term loans to enterprises, and strive to help enterprises broaden financing channels and strengthen the management of working capital loans.

Finally, although this study first proposed the U-shaped relationship between short-term debt and long-term debt and corporate default risk, there are still many deficiencies and limitations. Future research can continue to deepen on the basis of this article, such as further controlling the factors of China’s macroeconomic changes (fiscal policy, leverage factor, etc.). More importantly, the existing research does not consider the widespread phenomenon of short-term debt and long-term use of Chinese enterprises and its interaction with macro policy factors, which may lead to deviation of relevant conclusions, and even be detrimental to the national macro policy regulation. To sum up, in view of the long-term imbalance between the investment and financing term structure of China’s supply chain enterprises under the macroeconomic impact, we should not only play a regulatory role at the macro level, but also strengthen the internal management of enterprises at the micro level.

## Supporting information

S1 Data (XLSX)

## References

[1] Morris J.R. On Corporate Debt Maturity Strategies. J. Financ. 1976, 31, 29–37.

[2] Campello M, Giambona E, Graham JR, Harvey CR. Liquidity Management and Corporate Investment during a Financial Crisis. J. The Review of Financial Studies. 2011, 24, 1944–1979.

[3] Demerjian PR., Lev B., Lewis MF. Managerial ability and earnings quality. J. The Accounting Review. 2013, 88(2), 463–498.

[4] He JJ., Tian X. The dark side of analyst coverage: the case of innovation. J. Journal of Financial Economics. 2013, 109(3), 856–878.

[5] Kahl M, Shivdasani A, Wang Y. Short-Term Debt as Bridge Financing: Evidence from the Commercial Paper Market. J. Journal of Finance. 2015, 70(1), 211–255.

[6] Custódio C.; Ferreira M.A.; Laureano L. Why are US firms using more short-term debt? J. Financ. Econ. 2013, 108, 182–212.

[7] Zengfua Li, Junjieb Chen, Yujunc Lian and Mingjie Li. Economic Policy Uncertainty and Corporate Short-term Debt for Long-term Use. J. Journal of Management World. 2022, 38(01), 77–89+143+90–101.(in Chinese)

[8] Douglas W.D. Debt Maturity Structure and Liquidity Risk. J. Quarterly Journal of Economics. 1991, 106(3), 709–737.

[9] Acharya V.V.; Gale D.; Yorulmazer T. Rollover Risk and Market Freezes. J. Financ. 2011, 66, 1177–1209.

[10] Kanas A, Molyneux P. Do measures of systemic risk predict U.S. corporate bond default rates? J. International Review of Financial Analysis. 2020, 71.

[11] Myers S C. Determinants of Corporate Borrowing. J. Journal of Financial Economics. 1977, 5(02), 147–175.

[12] Nishi H. An empirical contribution to Minsky’s financial fragility: evidence from non-financial sector in Japan. J. Cambridge Journal of Economics. 2019, 43(3), 585–622.

[13] Francis B. B., Hasan I. and Zhu Y. Political Uncertainty and Bank Loan Contracting. J. Journal of Empirical Finance. 2014, 29, 281–286.

[14] Waisman M., Ye P. and Zhu Y. The Effect of Political Uncertainty on the Cost of Corporate Debt. J. Journal of Financial Stability. 2015, 16, 106–117.

[15] Jensen M.C., Meckling W.H. Theory of the Firm: Managerial Behavior, Agency Costs and Ownership Structure. J. Journal of Financial Economics. 1976, 3(4), 305–360.

[16] Rajan R. and Winton A. Covenants and Collateral as Incentives to Monitor. J. Journal of Finance. 1995, 50(4), 1113–1146.

[17] Khurana I.K., Wang C. Debt Maturity Structure and Accounting Conservatism. J. Journal of Business, Finance and Accounting. 2015, 42(1–2), 167–203.

[18] Flannery M.J. Asymmetric Information and Risky Debt Maturity Choice. J. Financ. 1986, 41, 19–37.

[19] Custodio C, Miguel F. and Luis L. Why are US Firms Using More Short-term Debt? J. Journal of Financial Economics. 2013, 108(1), 182–212.

[20] Gilchrist, S., Sim, J. W. and Egon, Z. Uncertainty Financial Frictions and Investment Dynamics. NBER Working Papers. 2014, No.20038.

[21] Nagar V., Schoenfeld J. and Wellman L. The Effect of Economic Policy Uncertainty on Investor Information Asymmetry and Management Disclosures. J. Journal of Accounting and Economics. 2019, 67(1), 36–57.

[22] Diamond D W. Debt Maturity Structure and Liquidity Risk. J. The Quarterly Journal of Economics. 1991, 106(03), 709–737.

[23] Kai Zhong, Deng Ya-Wen, Dong Xiao-dan. The Impact of Investment with Short-Term Financing on Corporate Risk. J. Journal of Management World. 2016(03):87–98+ 114+188.(in Chinese)

[24] Aghion P, Angeletos GM, Banerjee A, and Manova K. Volatility and Growth: Credit Constraints and the Composition of Investment. J. Journal of Monetary Economics. 2010, 57(03), 246–265.

[25] Guariglia A, Liu P. To What Extent Do Financing Constraints Affect Chinese Firm’s Innovation Activities. J. International Review of Financial Analysis. 2014, 36, 223–240.

[26] Lin JX., Sun X., Jiang Y. Endowment, industrial structure and appropriate financial structure: a new structural economics perspective. J. Journal of Economic Policy Reform. 2013(2), 109–122.

[27] Chen Y S., Shen C H., Lin C Y. The benefits of political connection: evidence from individual bank loan contracts. J. Journal of Finance Serve Research. 2014(45), 287–305.

[28] Cheng F., Chiao C., Fang Z., Wang C., Yao S. Raising short-term debt for long-term investment and stock price crash risk: Evidence from China. Financ. Res. Lett., 33 (2020), 101200. doi: 10.1016/j.frl.2019.05.018

[29] Xiaoguang LIU and Yuanchun LIU. Leverage, Short-term Debt for Long-term Use and Firm Performance. J. Economic Research Journal. 2019, 54(07), 127–141.

[30] Mclean DR, Zhao M. The Business Cycle, Investor Sentiment, and Costly External Finance. J. The Journal of Finance. 2014, 69(03), 1377–1409.

